# Body Composition of Young Women and The Consumption of Selected Nutrients

**DOI:** 10.3390/nu15010129

**Published:** 2022-12-27

**Authors:** Elżbieta Karpińska, Justyna Moskwa, Anna Puścion-Jakubik, Sylwia Katarzyna Naliwajko, Jolanta Soroczyńska, Renata Markiewicz-Żukowska, Katarzyna Socha

**Affiliations:** Department of Bromatology, Faculty of Pharmacy with the Division of Laboratory Medicine, Medical University of Białystok, Mickiewicza 2D Street, 15-222 Białystok, Poland

**Keywords:** BMI, body composition, body fat, young women, diet, dietary fiber, unsaturated fatty acids

## Abstract

The choices of food products and their nutritional value have a significant impact on nutritional status and body composition parameters. Popular and low-cost indicators of nutritional status, such as BMI, may not reflect the actual condition of the body. The aim of the study was to assess the consumption of energy as well as selected nutrients, such as protein, fats, carbohydrates, unsaturated fatty acids, lactose, starch, and dietary fiber, and to evaluate body mass index (BMI) and the parameters of the body composition among young women. Next, correlations were made between these nutrients and the parameters, such as percent body fat (PBF), visceral fat area (VFA), and fitness score (FS). The study group included 478 young women. To assess their consumption, the participants completed 72 h dietary interviews. In addition, the participants had a body composition analysis performed by bioelectric impedance technology by the InBody 720 Body Composition Analyzer (InBody, South Korea). The average energy value of the diets was about 1480 kcal. Most diets contained adequate portions of protein, fat, and carbohydrates. The diets were deficient in terms of unsaturated fatty acids and fiber. We showed that up to 37% of women with normal BMI had excess body fat. Moreover, the % energy from plant protein consumption negatively correlated with PBF and BMI (r = −0.10, *p* = 0.029, r = −0.10, *p* = 0.037, respectively) and dietary fiber with PBF, BMI, VFA, and FS (r = −0.17, *p* = 0.0003, r = −0.12, *p* = 0.01, r = −0.18, *p* = 0.0001, r = 0.09, *p* = 0.041, respectively). Education on the proper consumption of basic nutrients, including plant-based protein and dietary fiber, seems to be essential in preventing overweight and obesity.

## 1. Introduction

Overweight and obesity are defined as abnormal or excessive fat accumulation that poses health risks. Obesity is a major public health problem because there is a steady increase in the number of overweight people, and obesity carries a higher risk of other diseases [1]. The WHO’s 2022 report states that in 2016 1.9 billion adults aged 18 and older had a body mass index (BMI) over 25 kg/m^2^. This problem affects men more than women (63% vs. 54%) in Europe. The highest levels of both overweight and obesity are found in the Mediterranean and Eastern European countries [2]. A raised BMI is a major risk factor for noncommunicable diseases such as cardiovascular diseases [3], diabetes [4], and musculoskeletal disorders, especially osteoarthritis [5]. Excess body fat is related with some cancers such as endometrial, breast, ovarian, prostate, liver, gallbladder, kidney, and colon [6]. Another risk associated with obesity is nutritional deficiency. Life-course exposure to early undernutrition is often connected with eating highly processed food, which contains a very low level of nutritional elements, such as vitamins, mineral components, and polyphenols, and is high in fat, sugar, salt, and energy [7].

Nowadays, the proposed diets focus on changing the proportions of the basic ingredients (proteins, fats, and carbohydrates). This caused changes in body composition and had weight reduction effects in the fairly short period of observation of the experiment. Analyses of the long-term use of diets with different contents of carbohydrates, proteins, and fats show that high-protein diets have better results in the short term; within the space of months, the effect on the normalization of body weight is similar to that of the other diets [8]. Diet is supposed to provide the body not only with weight reduction but also with mental comfort and, above all, with the healthy nourishment of the body, which will prevent the diseases of civilization. 

The analysis of the diets of young people shows how the intake of individual nutrients correlates with the body composition parameters. Therefore, the aim of the study was to assess the consumption of energy and nutrients, such as protein, fats, carbohydrates, unsaturated fatty acids, lactose, starch, and dietary fiber, as well as to assess the BMI and selected body composition parameters among young women. In a further stage of the research, the relationship between the consumption of individual ingredients and body composition was assessed.

## 2. Materials and Methods

### 2.1. Study Group 

Five hundred people were invited to participate in the study; twenty-two were rejected due to formal deficiencies or health contraindications. The study group included 478 women aged 18 to 26 (Table 1). They were women mainly from the city of Bialystok and smaller towns in the Podlaskie region. The analysis of the questionnaires shows that most of the women surveyed were studying. 

The study did not place restrictions on the selection of the group in terms of various diets or special eating habits, which allowed the diet of the study group to be observed. Women between the ages of 18–26 are characterized by independence in choosing their dietary preferences. Young people, in most cases, do not have dietary restrictions due to their medical conditions. In the study, we limited ourselves to selecting one gender to make the statistical analyses more reliable with the assumed group size.

The study was approved by the Bioethical Committee of the Medical University of Bialystok no: R-I-002/39/2015. 

### 2.2. Estimating the Consumption of Nutrients

The individuals who qualified for the study completed 24 h dietary interviews for three days (two weekdays and one weekend day). This questionnaire is recommended by the Committee of Human Nutrition Science Polish Academy of Sciences in Warsaw [9]. The size of the consumed portion of food was assessed using the “Photo album of products and dishes” [10]. The nutritional interviews were analyzed by the computer program Diet 6.0, which was developed in the Independent Laboratory of Epidemiology and Nutrition Standards of the Institute of Food and Nutrition in 2018 (Warsaw, Poland). This program calculated all the nutrients (such as calories, carbohydrates and carbohydrate fractions, protein, fat and individual fatty acids, and others). The obtained values of nutrients were compared with the Polish recommendations [11], which are in most cases comparable to the reference nutritional values for nutrients issued by the European Food Safety Authority (Table 2) [12].

The percentage of energy from the proteins, fats and carbohydrates, liloleic acid, and alpha-linolenic acid was calculated. The eicosapentaenoic acid (EPA), docosahexaenoic acid (DHA), liloleic acid (LA), alfa-linoleic acid (ALA), lactose, starch, and dietary fiber were calculated by weight (gram/day). Then, the consumption was compared to the norms. 

### 2.3. Body Composition Analysis

All study participants underwent body composition analysis using the In Body 720 Body Composition Analyzer (InBody, South Korea). This device uses bioelectrical impedance technology. The InBody 720 has 8 electrodes, including 2 electrodes for one limb. This analysis uses up to 6 bioimpedance frequencies (1, 5, 50, 250, 500, and 1000 kHz). The 5-cylinder technology analyzes each limb and trunk separately. 

The women began the analysis fasting. An additional condition was the need not to undertake additional physical activity on the previous day.

The analyzer determines body fat, lean tissue, percentage of body fat (PBF), visceral fat area (VFA), and fitness score (FS). InBody indicates that underweight women are those with a PBF below 18%. Women with a PBF between 18 and 28% were included in the group with a normal body fat content, while those with a PBF above 28% were included in the group of overweight and obese women.

VFA is a tissue that is accumulated around the organs; its content is expressed in cm^3^. For young people, the maximum allowable content is 100 cm^3^.

The FS takes into account the content of muscle mass and body fat. This indicator increases with increasing muscle mass. It can take values below 70 (weak build, obese person), from 70 to 90 (normal build, healthy person), or above 90 (strong, muscular type).

The BMI index was assessed according to the standards of the WHO [13]: below 18.5 kg/m^2^, underweight; 18.5–24.9, normal weight; 25.0–29.9, overweight; 30.0–34.9, obesity class I; 35.0–39.9, obesity class II; above 40.0, obesity class III. 

### 2.4. Statistical Analysis

The Statitistica V 13.3 computer program was used for statistical analysis. Values with *p* < 0.05 were considered statistically significant. The normality of the distribution was assessed using the Kruskal–Wallis and the Liliefores test—the lack of normality of the data distribution was demonstrated. Spearman’s rank correlation for non-parametric variables measures was used. R indicates the strength and direction of association between two ranked variables.

## 3. Results

The median energy value of the diets was 1480 kcal (Q1: 1221, Q3: 1810). The median protein intake was 69.0 g, including 21.7 g of vegetable protein and 44.9 g of animal protein. In the study group, the consumption of fat was 45.0 g and the consumption of carbohydrates was 203.4 g. Other values describing the consumption of individual components are presented in Table 3.

Most of the study group did not consume enough energy (85.5%), but the percentages of people with an insufficient percentage of energy from protein, fats, and carbohydrates were: 33.5%, 29.1%, and 30.1%, respectively. It is worth emphasizing that the percentage of people with an insufficient intake of linoleic acid was 75.9%, and for the dietary fiber, it was as much as 87.9% (Table 4).

The median BMI in the examined group of young women was 21.05 kg/m^2^; the content of visceral tissue was 52.3 cm^2^; and the percent body fat was 26.5% (Table 5).

**Figure 1:**
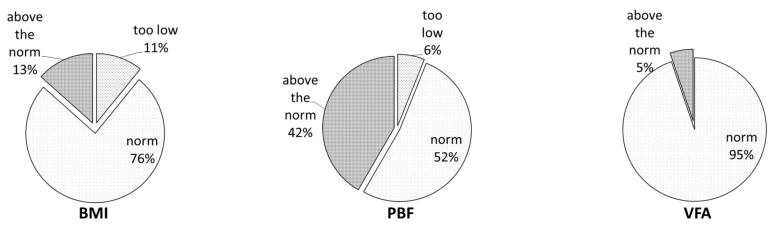
Percentage of women depending on the value of the body mass index (BMI), percent body fat (PBF), and visceral fat area (VFA).

We showed that the majority of the examined women had a normal BMI (76%); 11% were underweight; and 13% were overweight or obese. In turn, the analysis of the percentage of visceral tissue showed that up to about 42% of the women had a tissue content above the norm, i.e., above 28%. The vast majority of the women, i.e., over 94.6%, had normal visceral fat content. Only 5.4% of the women had this parameter above normal (Figure 1).

Further analysis showed that among the women with a normal BMI (76%), only 4% had an insufficient amount of adipose tissue, and up to 37% had an excessive percentage of this tissue.

The Spearman’s rank correlation analysis between the amount of consumed energy and nutrients and the parameters of the body composition analysis showed significant relationships, which are presented in Table 6.

It was shown that with an increase in the BMI and the content of adipose tissue, both the subcutaneous and the visceral, the % of energy from the proteins increases, but the % of protein of plant origin decreases significantly (Table 6).

The analyzed parameters of the body composition are also dependent on the consumption of dietary fiber, starch, and lactose. The correlation results showed that higher BMI and body fat content were associated with lower fiber and starch intake.

The FS scale is related to the consumption of lactose and dietary fiber. People with a higher intake had better body composition parameters (Table 6). 

We noted significant differences in energy content, % energy from protein, % energy from animal protein, starch, and dietary fiber between groups of women with different body fat (PBF) (Table 7).

## 4. Discussion

Body composition analyses are useful tools for assessing the effectiveness of nutritional interventions and monitoring the changes associated with a disease condition such as obesity. With a good standardization of the methods, instruments, and preparation of the individuals, body mass composition analysis can provide a quick and easy assessment of fat, lean mass, and other parameters in healthy populations and in obese individuals [14]. 

In our study, the comparative analysis of the BMI and body composition results shows that more than 1/3 of the surveyed women with normal BMIs, according to the body composition parameters, should be classified as people with an excessive amount of adipose tissue. That is, in the entire surveyed population, 28% of the people were incorrectly classified. Other authors confirm that the BMI indices do not reflect the correct PBF values [15].

The correlation analysis showed that increased caloric content in the nutritional intervention decreases the BMI and the concentration of fat tissue (Table 6). The important fact is that almost all of the analyzed diets had energy below the recommended standard (Table 4). This may be because of people with poorer body composition parameters; they often underestimate the caloric content of the portions consumed. Underestimating caloric content is more common in women with higher education than other population groups [16]. Other studies have shown that estimations of the caloric content of an unhealthy main-meal food tend to be lower when the food is shown alongside a healthy item (e.g., fruit or vegetables) than when shown alone [17,18]. The unhealthy foods that one underestimates are, for example, high-calorie drinks [19].

The strategies to treat obesity have been focused on significant lifestyle modifications, including diets [20]. High-protein, ketogenic, vegan, or gut microbiota-altering diets are often promoted [21]. Their effectiveness is being considered in terms of both short-term and long-term follow-up [22]. Lifestyle is one of the main factors affecting obesity; therefore, it is necessary to study the eating habits of young people in correlation with body composition. 

**Figure 2:**
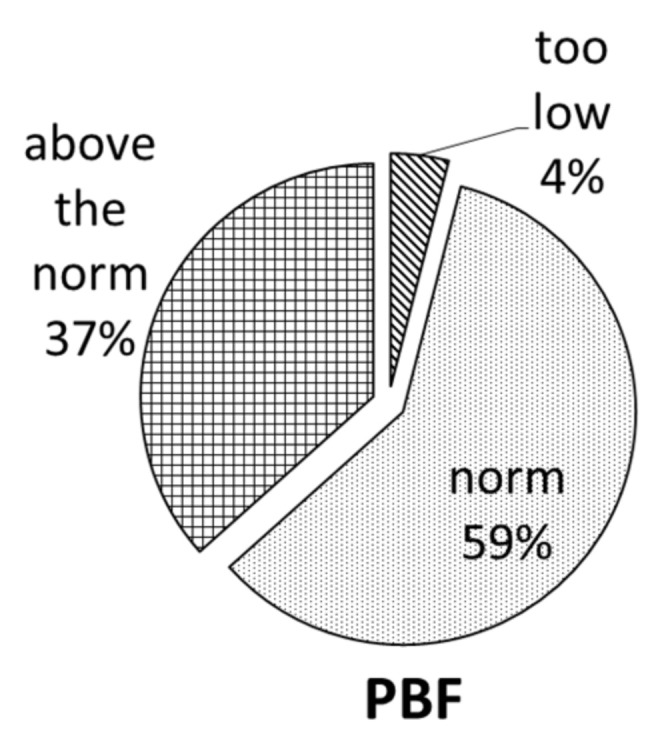
Percentage of women with normal BMI depending on percent body fat (PBF).

In the present study, the focus on the group of young women and BMI indicates that 76% of them were of normal weight and that 13% of the women had a BMI above 25 (Figure 1). The results show that among the entire study group, 42% of the women had excessive body fat (BF) (Figure 1) and that among those with a normal BMI, up to 37% of the participants were shown to have excessive subcutaneous fat (Figure 2). This may indicate that almost 29% of the young women surveyed, aged 19–26, have so-called normal-weight obesity (NWO). NWO is defined as having a normal BMI but a high body fat mass [23,24]. A similar study, conducted by Čuta et al. (2019) [25], showed that up to 14% of the examined young Czech women (aged 20-30) were found to have NWO. The research indicates that NWO among young people is associated with increased cardiometabolic, diabetes, and subclinical atherosclerosis risk [26,27,28]. The percentages of proteins, fats, and carbohydrates in the diets of most of the studied young women were normal, but deficiencies were observed for nutrients such as essential fatty acids, including LA, ALA acid, and the sum of EPA and DHA, and dietary fiber (Table 4). Numerous scientific publications indicate that the diet of young women is deficient as far as DHA intake is concerned [29,30]. Wierzejska et al. [31] showed that the DHA intake by pregnant women was about 60 mg/day. Overall, 92% of the subjects consumed <200 mg of DHA a day, which was the result of insufficient fish consumption (mean: 15 g/day). The scientific reports from European countries indicate that about half of the countries have sufficient linoleic acid intake (4% of energy) and that adolescents and young women are more likely to have insufficient intake. The mean ALA intake was within the recommendation (0.5 % energy) in 77% of the European countries. In 26% of the countries, the mean EPA and/or DHA intake was as recommended [32]. Sioen et al. [33] showed that in Belgian children, fats and oils were the major contributors to the intakes of LA (23.6%) and ALA (33.1%), followed by cereal products with 17.6 and 13.5%, respectively. The multiple country HELENA study [34] indicated that the food group “meat, fish, eggs and meat alternatives” was the largest contributor to the intake of LA (31.7%), ALA (21.5%), arachidonic acid (ARA, 54.2%), EPA (92.3%), docosapentaenoic acid (DPA, 94.9%), and DHA (85.8%) in adolescents. The study showed that fats and oils were the main contributor to the LA (39%) and ALA (36%) intake, whereas fish and shellfish (29%) and meat and meat products (28%) were the main contributors to the EPA and DHA intake in the elderly [32]. The insufficient intake of omega-3 fatty acids causes a high risk of inflammation in the body and the onset of disorders, including depression [35,36]. Dietary fiber has been a very important part of healthy diets, but the research indicates that its intake is too low in the European population. Seljak et al. [37] showed that the percent of the population with inadequate fiber intakes (<30 g/day) was 90.6% in adolescents, 89.6% in adults, and 83.9% in the elderly, while the mean daily fiber intakes were 19.5, 20.9, and 22.4 g, respectively. The significant determinants for inadequate dietary fiber intake were sex in adolescents and adults and BMI in adults. The main food groups contributing to dietary fiber intake were bread and other grain products, vegetables, and fruits, with significant differences between population groups. The contribution of fruits and vegetables to the mean daily dietary fiber intake was highest in the elderly (11.6 g), followed by adults (10.6 g) and adolescents (8.5 g). In contrast, dietary fiber intake in the elderly female population in Europe is only 3–8 g/day [38]. Studies by other authors indicate that dietary fiber influences energy intake and reduces the risk of the development of obesity. Dietary fiber improves anthropometric and metabolic outcomes in adults, such as a reduction in BMI, body weight, and body fat mass and fasting glucose, and fasting insulin [39,40]. The scientific research has shown that the consumption of starch is associated with reduced abdominal fat and improved insulin sensitivity [41,42,43]. In this study, we showed that the dietary fiber intake (Table 4) was insufficient in the entire group. It should be emphasized that more than 40% of the surveyed women had a PBF above the norm. The correlation results showed that higher BMI and body fat content were associated with lower fiber intake. Other authors indicate that dietary fiber intake improves body composition, reduces overweight, and facilitates body balance. The scientific studies confirmed that dietary fiber affects less weight gain, which may be associated with its positive effect on the microbiota [8,44].

A positive correlation between the consumption of lactose (contained in milk products) and a better fitness score in the body composition analysis was observed in our study (Table 6). Comparing the results of other authors, there was a significant increase in the consumption of milk and dairy products and lean body mass. The effectiveness of consuming milk and dairy products in increasing lean body mass is more pronounced in the participants with a baseline body weight within the normal range than in those who are overweight or obese [45,46,47,48,49]. In the current study, a positive correlation was observed between protein intake and an increase in BMI, PBF, and VFA in the studied young women, while a BMI and PBF decrease with an increase in plant protein intake was observed (Table 6). Several studies have shown that soy protein interventions in overweight and obese individuals decrease body mass, waist circumference, and BMI [50]. Haghighat et al. [51] showed that six months of a soy-enriched high-protein snack replacement decreased appetite and improved body composition in women with NWO. The other meta-analysis on young women indicates that soy products have weight-loss effects, mainly due to soy protein, isoflavone, and soy fiber [52]. 

We are aware that our study has some limitations, one of which was that it was not randomized. According to the Strengthening the Reporting of Observational Studies in Epidemiology guidelines [53], this was an observational case–control study. The methods used to assess diet have their imperfections. A defect of the 24 h dietary interview is the underestimation of the energy intake data. This trend is gender-related, being stronger among women. However, the strongest determinant of the underreporting of energy intake is BMI. People with a higher BMI are more likely to underestimate the energy value of the food they consume. Moreover, we can conclude that socially desirable foodstuffs were not underrated to the same extent as socially undesirable products—similar conclusions have also been made by other authors [54].

## 5. Conclusions

BMI, with respect to the analyzing of the nutritional status of the body, is too weak a parameter. According to our study, 28% of participants were classified as obese according to the body composition and misclassified as the normal weight with the BMI. Despite the fact that the majority of the group (76%) have a normal BMI, according to the assessment of body fat content, about half of the population has a normal PBF. Excessive body fat may be influenced by the insufficient intake of dietary fiber and starchy foods. The insufficient intake of EFAs has also been noted in the population. There is a noticeable trend indicating that people with better body composition parameters consume more plant protein. Future studies with a wider group and the male gender are needed to confirm this conclusion.

## Figures and Tables

**Table 1:** Characteristics of study group.

Parameter	Av. ± SD Min–Max	Med Q1–Q3
Age (years)	21.1 ± 1.5 18.0–26.0	20.0 20.0–22.0
Weight (kg)	60.9 ± 10.4 38.8–104.8	59.5 53.6–66.4
Height (cm)	167.3 ± 6.1 150.0–187.0	167.0 163.0–171.0

Av.—average, IQR—interquartile range, Max—maximum value, Med—median, Min—minimum value, Q1—lower quartile, Q3—upper quartile, SD—standard deviation.

**Table 2:** Dietary reference values of nutrients for young women aged 19–30 years old.

Energy and Components of Diet	EFSA Recommendation [12]	Nutrition Standards for the Polish Population [11]
Energy (kcal)	-	2050–2300
% energy from protein	-	15–20
% energy from fat	20–35	20–35
% energy from LA	4	4
% energy from ALA	0.5	0.5
% energy from carbohydrate	45–60	45–65
EPA + DHA (mg/day)	250	250
Dietary fiber (g/day)	25	25
Lactose (g/day)	-	-
Starch (g/day)	-	-

ALA—alfa-linoleic acid, EFSA—European Food Safety Authority, EPA + DHA—eicosapentaenoic and docosahexaenoic fatty acids, LA—liloleic acid.

**Table 3:** Consumption of selected nutrients.

Parameter	Av. ± SD Min–Max	Med Q1–Q3
Energy (kcal)	1554 ± 509 583–4708	1480 1221–1810
Total protein (g)	70.5 ± 19.5 27.5–146.0	69.0 56.2–81.8
Animal protein (g)	46.9 ± 16.3 3.7–113.2	44.9 34.0–56.4
Plant protein (g)	22.5 ± 8.0 5.9–88.0	21.7 17.4–26.9
Fat (g)	48.2 ± 27.3 8.2–361.8	45.0 32.4–57.2
Total carbohydrates (g)	217.8 ± 80.0 59.2–611.7	203.4 166.3–250.8
Saturated fatty acids (g)	18.9 ± 12.4 2.1–168.1	16.9 12.5–22.9
Monounsaturated fatty acids (g)	17.9 ± 10.3 2.8–117.6	16.4 11.4–21.6
LA (g)	5.83 ± 3.66 1.52–43.02	5.03 3.61–7.13
% of energy from LA	3.34 ± 1.46 1.21–11.87	3.05 2.36 -3.98
ALA (g)	1.10 ± 0.90 0.13–9.58	0.84 0.55–1.30
% of energy from ALA	0.63 ± 0.47 0.13–6.05	0.49 0.36–0.75
EPA + DHA (mg/d)	198.4 ± 433.2 0.0–4523.9	57.3 29.1–131.8
PUFA (g)	7.30 ± 4.37 1.76–49.03	6.32 4.64–8.92
Cholesterol (mg)	252.7 ± 128.4 1.6–1125.1	234.6 165.0–313.3
Saccharose (g)	35.5 ± 24.3 2.5–184.0	28.9 18.7–46.1
Lactose (g)	9.02 ± 5.61 0.00–26.94	7.94 4.81–12.41
Starch (g)	109.5 ± 39.2 16.1–300.4	107.6 83.7–133. 8
Dietary fiber (g)	17.6 ± 7.3 3.0–54.1	16.3 13.0–20.6
LC-PUFA (g)	0.223 ± 0.492 0.000–5.207	0.061 0.032–0.151
Absorbable carbohydrates (g)	200.2 ± 74.9 54.5–574.4	187.7 153.0–231.7
% energy of the diet of digestible carbohydrates	51.4 ± 8.1 16.6–73.47	51.4 46.1–56.5
% energy from protein	18.7 ± 4.0 6.7–35.9	18.3 16.0–21.0
% energy from fat	27.3 ± 7.4 10.6–75.1	27.0 22.7–31.5
% energy from carbohydrates	51.7 ± 8.1 17.0–73.6	51.6 46.4–56.9

ALA—alfa-linoleic acid, EPA + DHA—eicosapentaenoic and docosahexaenoic fatty acids, LA—liloleic acid, PUFA—polyunsaturated fatty acids, LC-PUFA—long-chain polyunsaturated fatty acids.

**Table 4:** Percentage of people with sufficient and insufficient intake of energy and selected ingredients.

Energy and Components of Diet	Percentage of People with Sufficient Intake (%)	Percentage of People with Insufficient Intake (%)
Energy	14.2%	85.8%
% energy from protein	66.5%	33.5%
% energy from fat	70.9%	29.1%
% energy from LA	24.1%	75.9%
% energy from ALA	47.9%	52.1%
EPA + DHA	16.5%	83.5%
% energy from carbohydrate	69.9%	30.1%
Dietary fiber	12.1%	87.9%

ALA—alfa-linoleic acid, EPA + DHA—eicosapentaenoic and docosahexaenoic fatty acids, LA—liloleic acid.

**Table 5:** Characteristics of the body composition of the studied women.

Parameter (Unit)	Av. ± SD Min–Max	Med. Q1–Q3
BMI (kg/m^2^)	21.71 ± 3.21 15.30–38.04	21.05 19.57–23.2
Body fat mass (kg)	16.82 ± 6.58 4.00–49.60	15.55 12.10–19.80
Fat-free mass (kg)	55.8 ± 23.1 5.0–40.7	43.55 40.30–47.30
PBF (%)	26.9 ± 6.3 9.2–48.5	26.5 22.4–31.0
VFA (cm^3^)	44.07 ± 5.40 30.20–61.20	52.3 40.7–67.0
FS	73.0 ± 4.9 54.0–88.0	73.0 70.0–76.0
Lean mass of right arm (%)	95.59 ± 14.23 61.79–148.60	94.55 86.00–103.90
Lean mass of left arm (%)	93.79 ± 14.53 59.50–148.20	92.72 84.28–101.70
Lean mass of trunk (%)	95.64 ± 8.94 74.80 -132.60	94.60 89.20–101.00
Lean mass of left leg (%)	100.64 ± 8.81 76.70–131.60	100.20 95.00-106.20
Lean mass of right leg (%)	100.72 ± 8.91 77.2–133.80	100.4 94.95–106.32
Basal Metabolic Rate (kcal)	1321.8 ± 116.7 1023.2–1691.7	1310.5 1241.0–1391.0

BMI—body mass index, FS—fitness score, PBF—percentage of body fat, VFA—visceral fat area.

**Table 6:** Correlation between parameters of the body composition and consumed energy and components of diet (*n* = 478).

Energy and Components of Diet	Body Mass Index		Percent Body Fat		Visceral Fat Area		Fitness Score	
	*p* Value	R Spearman	*p* Value	R Spearman	*p* Value	R Spearman	*p* Value	R Spearman
Energy (kcal)	<0.0001	–0.24	<0.000	–0.21	<0.0001	–0.18	-	-
% energy from protein	<0.0001	0.17	0.021	0.11	0.024	0.10	-	-
% energy from plant protein	0.037	–0.10	0.029	– 0.10	-	-	-	-
% energy from animal protein	-	-	0.044	0.10	-	-	-	-
Lactose (g)	-	-	-	-	-	-	0.022	0.11
Starch (g)	<0.0001	–0.25	<0.0001	–0.24	<0.0001	–0.18	-	-
Dietary fiber (g)	0.010	–0.12	0.0003	–0.17	0.0001	–0.18	0.041	0.09

**Table 7:** Comparison of nutrient intake by study group: Group 1—women with percent body fat (PBF) below 18%; Group 2—women with PBF between 18 and 28%; Group 3—women with PBF above 28%.

Energy And Components of Diet	Group 1	Group 2	Group 3	
	Med. Q1–Q3	Med. Q1–Q3	Med. Q1–Q3	*p* Value
Energy (kcal)	1884.0 1598.1–2206.1	1514.3 1284.7–1837	1369.0 1148.0–1707.0	<0.0001
% energy from protein	17.0 15.8–18.1	18.0 15.8–24.0	19.0 16.7–21.7	0.003
% energy from plant protein	5.69 5.41–6.55	5.71 5.02–6.56	5.79 5.08–6.53	---
% energy from animal protein	10.1 9.13–12.2	12.1 9.75–14.3	13.0 10.4–15.8	0.001
Starch (g)	141.7 117.9–161.9	110.9 90.0–137.1	99.5 75.6–123.1	<0.0001
Dietary fiber (g)	16.8 14.9–24.2	16.8 13.4–21.1	15.2 12.0–19.6	0.003

Med—median, Q1—lower quartile, Q3—upper quartile.

## Data Availability

Detailed data are available from the authors.
